# Promising Plant-Derived Adjuvants in the Development of Coccidial Vaccines

**DOI:** 10.3389/fvets.2019.00020

**Published:** 2019-02-12

**Authors:** Valeria A. Sander, Mariana G. Corigliano, Marina Clemente

**Affiliations:** Unidad de Biotecnología 6-UB6, Instituto Tecnológico Chascomús (INTECh), Consejo Nacional de Investigaciones Científicas y Técnicas (CONICET)-Universidad Nacional de General San Martín (UNSAM), Chascomús, Argentina

**Keywords:** coccidial parasites, plant-derived adjuvants, saponins, polysaccharides, lectins, heat shock proteins, vaccines

## Abstract

Coccidial parasites cause medical and veterinary diseases worldwide, frequently leading to severe illness and important economic losses. At present, drugs, chemotherapeutics and prophylactic vaccines are still missing for most of the coccidial infections. Moreover, the development and administration of drugs and chemotherapeutics against these diseases would not be adequate in livestock, since they may generate unacceptable residues in milk and meat that would avoid their commercialization. In this scenario, prophylactic vaccines emerge as the most suitable approach. Subunit vaccines have proven to be biologically safe and economically viable, allowing researchers to choose among the best antigens against each pathogen. However, they are generally poorly immunogenic and require the addition of adjuvant compounds to the vaccine formulation. During the last decades, research involving plant immunomodulatory compounds has become an important field of study based on their potential pharmaceutical applications. Some plant molecules such as saponins, polysaccharides, lectins and heat shock proteins are being explored as candidates for adjuvant/carriers formulations. Moreover, plant-derived immune stimulatory compounds open the possibility to attain the main goal in adjuvant research: a safe and non-toxic adjuvant capable of strongly boosting and directing immune responses that could be incorporated into different vaccine formulations, including mucosal vaccines. Here, we review the immunomodulatory properties of several plant molecules and discuss their application and future perspective as adjuvants in the development of vaccines against coccidial infections.

## Introduction

The phylum Apicomplexa is a large group of obligate intracellular protozoan parasites, comprising more than 6,000 species (1), characterized by the presence of an assembly of organelles called the apical complex (2) with some members being causative agents of the most life-threatening infectious diseases of humans and other animals (3–6), even contributing to increase human poverty (7, 8). In particular, among the most devastating Apicomplexan parasites are those referred to as “coccidial parasites” such as *Toxoplasma gondii, Cryptosporidium parvum, Cyclospora cayetanensis, Neospora caninum, Eimeria* spp., and *Isospora* spp. (9). In fact, *T. gondii*; *C. parvum*, and other *Cryptosporidium* species are not only a major public health concern causing severe human disease (10–12), but also cause significant economic damage to the livestock industry (13–15). In the same way, parasites including *N. caninum* and several species of the genus *Eimeria* and *Isospora* have been reported to have an important negative impact on economic animal production and animal welfare (16–22), leading to global annual estimated losses in cattle industries exceeding US $1.300 million (16) and in poultry production industry in excess of US$ 2 billion (22).

The outstanding ability of most coccidial parasites to invade multiple vertebrate hosts and effectively manipulate their immune responses, represent a huge challenge to most currently available control strategies. Despite considerable efforts have been made during the last decades to develop effective prophylactic as well as therapeutic drugs and vaccines, there has been only limited progress. Most of the drugs developed against coccidial parasites are poorly effective or cause several side effects (10, 11, 13, 23, 24) and when effective drugs have been identified, as in the case of avian coccidiosis, resistance frequently develops quickly (25, 26). Besides, there is an increased public concern about the use of chemotherapeutics in livestock, since they may generate unacceptable residues in milk and meat that would avoid their commercialization and consumption (27). Moreover, many anti-coccidial drugs are being banned from use in food animals (26). Although prophylactic vaccines emerge as the most suitable approach, successful vaccines against coccidial parasites are scarce and limited to the veterinary field (28). Most of them belong to one of the following categories: live attenuated vaccines, killed vaccines or subunit (and recombinant) vaccines. Currently, excluding one subunit vaccine against chicken coccidiosis (Coxabic) [reviewed in (29)], commercial vaccines against coccidial parasites are based on live virulent or attenuated organisms and whole killed organisms [reviewed in (26, 28)]. However, the safety of live vaccines is questionable due to the risk of virulence reversion (30). In contrast, recombinant subunit vaccines offer safer alternatives (30) and may provide the best long-term sustainable solution. However, purified antigens derived from different host systems are often less antigenic and immunogenic than attenuated or killed vaccine versions and the major challenge ahead is to devise effective ways to deliver these antigens to the immune system in order to stimulate appropriate immune responses (31, 32). In this regard, it is widely accepted that subunit vaccines require additional components to improve protective immunity. These components are molecules, compounds, or macromolecular complexes known as adjuvants (31). When incorporated into a vaccine formulation, adjuvants are capable of enhancing the magnitude of an adaptive response or modulating it toward the desired immune response to produce the most effective forms of immunity against each pathogen (31, 32). Despite during the last decades several adjuvants have been assayed in experimental subunit vaccines against coccidial parasites (11, 33), none of them have demonstrated to effectively protect against parasite infection, thus search for more appropriate and effective adjuvants is still one of the main challenges in the development of coccidial vaccines. Among the novel proposed adjuvants, plant-derived molecules such as saponins, polysaccharides, lectins, and heat shock proteins have proven to be potent immune stimulatory compounds with low toxicity and side-effects (34, 35). In this review we will explore the most promising plant-derived adjuvant molecules, and based upon their main immune effects and proposed mechanism of action, we will discuss their potential application in the development of new generation vaccines against coccidial parasites.

## Adjuvants: The Black Box of Immunology Being Opened

### General Considerations

**Figure 1:**
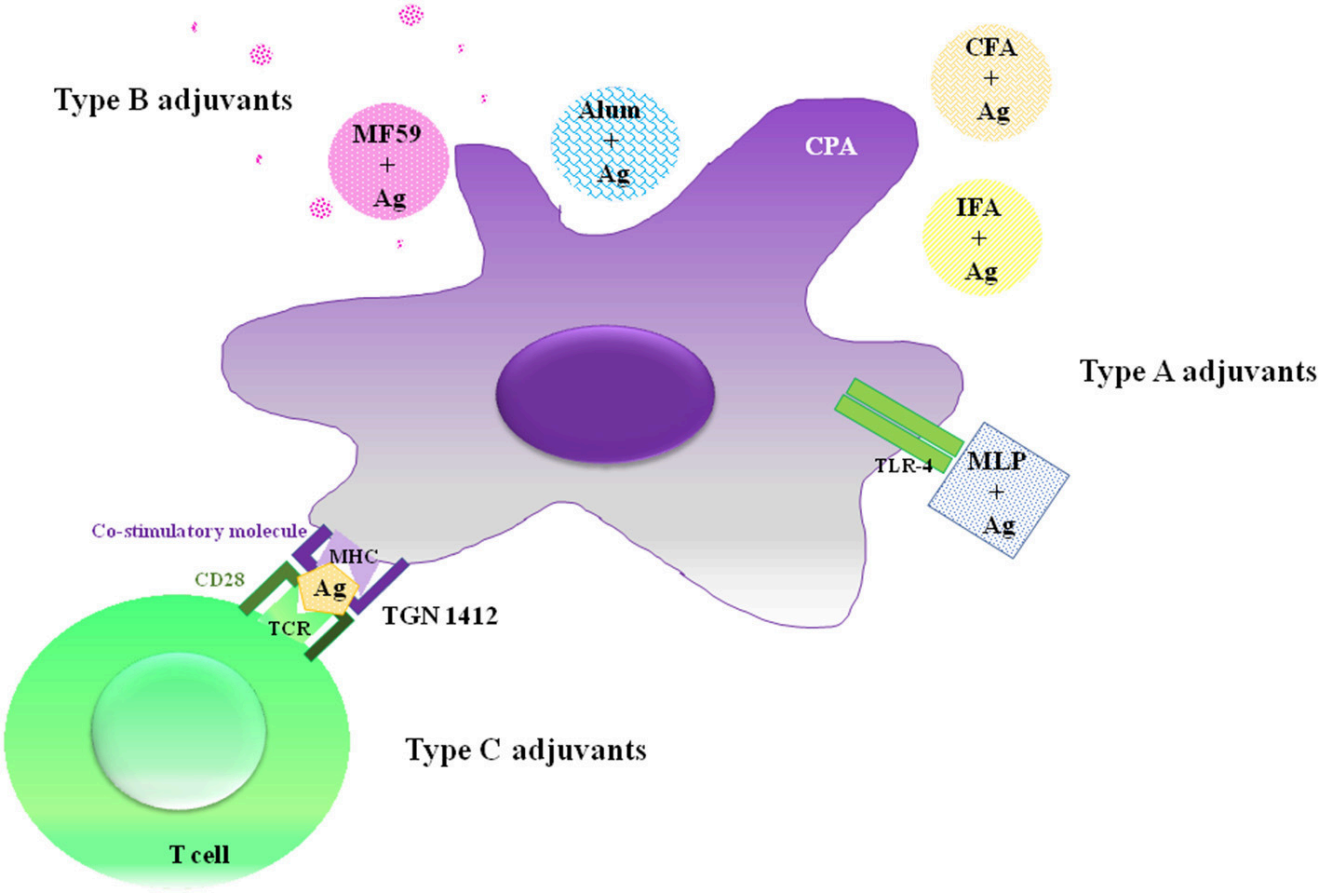
Classification of adjuvants. Type A adjuvants interact with pattern recognition receptors (such as Toll like receptors) and act as immune-potentiators of the immune response (e.g., MPL). Type B adjuvants function as delivery systems by improving the recruitment of innate immune cells and favoring the Ag capture (e.g., Alum, MF59, CFA, IFA). Type C adjuvants are those compounds that act through co-stimulatory molecules (CD28) present in T cells (e.g., TGN1412).

Vaccine adjuvants are used to improve the potency of the immune response to co-administered antigens. Most adjuvants are chemicals, molecules or particles obtained from infectious agents or their derived toxins (e.g., FCA, monophosphoryl lipid A, CT-B), mammalian proteins involved in “danger signals” and even molecules or proteins from plants with immunomodulatory properties (31, 34). Although adjuvant mechanisms of action are still unclear, in the last 20 years significant progress has been made to identify them. Three types of adjuvants are generally recognized depending on their effector mechanisms: Type A adjuvants (e.g., monophosphoryl lipid A, CpG ODN), which are Pattern Recognition Receptor (PRR) agonists (36, 37); type B adjuvants (e.g., Alum hydroxide, MF59, Freund's adjuvant, toxin-derived adjuvants, nanoparticles), which interact with antigen presenting cells (APCs) and antigens in an unspecific manner, which is the so-called depot effect, and associate with the antigen to facilitate its transport to the lymph node (37, 38); and, the less explored type C adjuvants, which are compounds that interact with co-stimulatory molecules on APCs (e.g., CD28 superagonist antibody TGN1412) (37) (Figure 1). Recently, an interesting approach arose to better understand the mechanisms of action of adjuvants based on the search for their molecular and cellular signatures (39–41). In this sense, genome-wide transcript microarray analysis has demonstrated that CpG ODN, an oligonucleotide previously characterized as a type A adjuvant, can modulate an adaptive immune response and regulate a large number of MF59 (type B adjuvant)-responsive genes, suggesting that the effector mechanisms of adjuvants are far from being completely elucidated (39) and must be evaluated *in vivo*, in a more global way. Indeed, any classification of adjuvants is difficult and may be incomplete, thus many of them resist easy definitions.

Although the rational design of vaccines against each pathogen has its own difficulties to overcome and represents singular challenges, there are some common features that should be taken into account in the selection of an adjuvant for the development of vaccine formulations against coccidial parasites. It is generally accepted that an appropriate immune response against intracellular obligate protozoans is primarily dependent on the cellular immunity mediated by both CD4^+^ and CD8^+^ T cells and their ability to secrete cytokines such as IFN-γ, as in the case of *T. gondii* (42), *Eimeria* spp. (43), *N. caninum* (33), *Cryptosporidium* spp. (44), and *I. suis* (21). These results suggest that a candidate adjuvant for a potential anti-coccidial vaccine should allow the correct processing and presentation of antigen to the host immune system to stimulate proper cell mediated immune responses with reduced toxicity (26). In addition, it would be highly recommended to deliver the vaccine through the natural site of entry of most coccidial parasites, the gut, so the development of an adjuvant that could be orally/intranasally administered would also be desirable (32, 45). In fact, among the various routes for application of vaccines, mucosal immunizations depict many attractive features over the parenteral routes, including lower risk of reactogenicity (32, 45). However, most of the currently available mucosal vaccines (or those in clinical trials) contain adjuvants that cause several side effects [reviewed in (45)], which encourage the search for new and safer mucosal adjuvants.

In the last decades, research involving plant immunomodulatory compounds has become an important field of study (34, 46). One of their most remarkable characteristics rely on their capability of eliciting proper immune responses with reduced toxicity (35, 46), and even some of them are able to act as antigen carriers (35) or to deliver the antigens to M cells in the Peyer's Patch (47). Among the most promising plant molecules and proteins for the development of adjuvants are saponins, polysaccharides, lectins and heat shock proteins, thus their properties and potential inclusion in anti-coccidial vaccines will be discussed in the following sections.

### Saponins and Saponin-Derived Compounds

**Figure 2:**
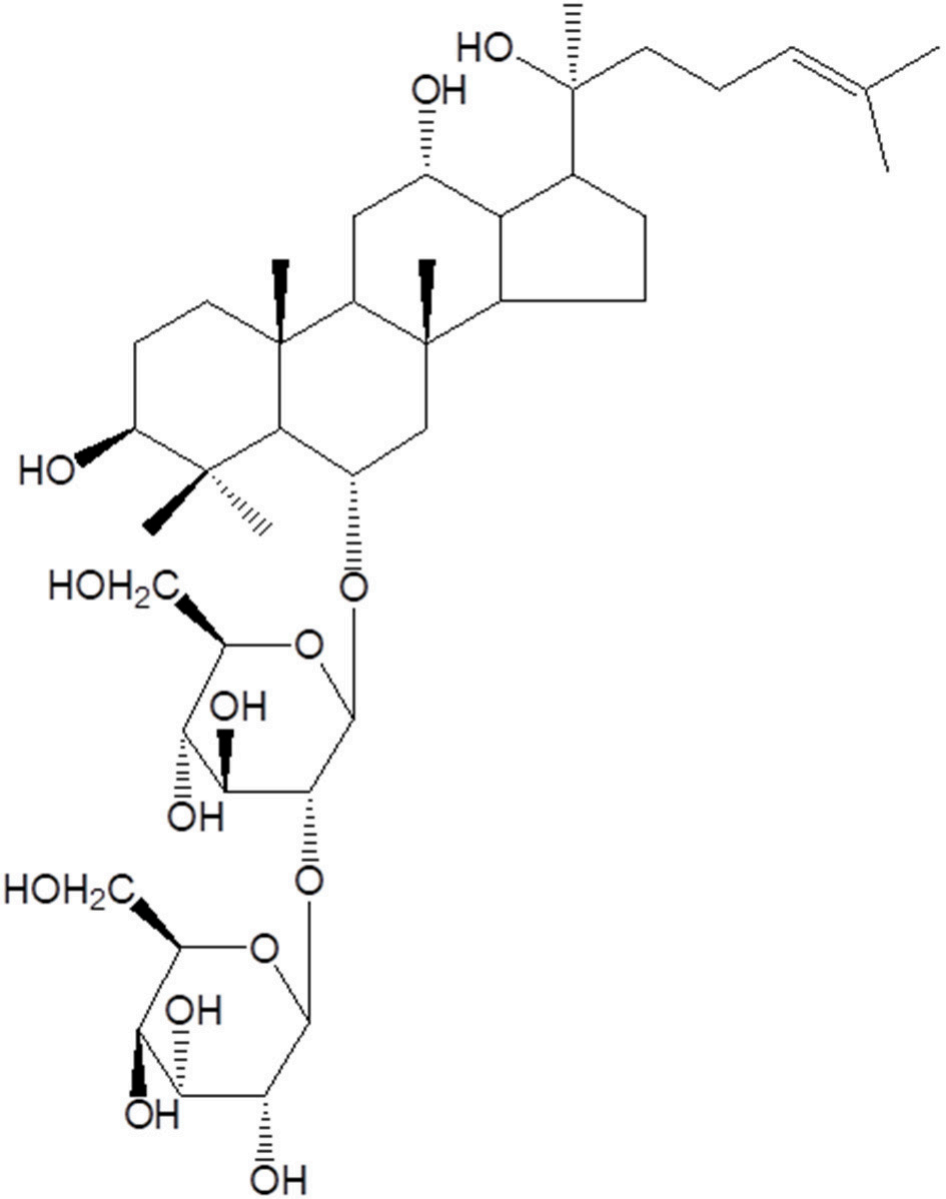
Molecular structure of Rg2 saponin from traditional Chinese medicinal herbs with adjuvant activities.

Saponins are natural steroidal or triterpene glycosides with immune modulatory properties (48–52), widely distributed in higher plants and usually found in roots, tubers, stems, barks, leaves, blooms and seeds (53). Basically, their chemical structures consist in non-polar aglycones linked to one or more carbohydrate chains (Figure 2).

In general, plants produce saponins during their normal development. However, Sparg et al. (53) suggested that saponins are involved in plant defense mechanisms due to anti-microbial, fungicidal and insecticidal activities. In addition, several plant saponins are able to activate the mammalian immune system, leading to significant interest in their potential as vaccine adjuvants (Table 1). In fact, Quil A is a partially purified mixture of saponins obtained from *Quillaja saponaria* and represents the most widely used and studied saponin-based vaccine adjuvant in mammals (37). Hence, Quil A and its purified form, saponin QS-21, have long been used as adjuvants in veterinary vaccines (82, 83). Quil A stimulates both humoral and cellular responses against co-administered antigens, with the generation of Th1 and cytotoxic T lymphocytes (CTLs) responses (37). The ability to elicit this type of immune response makes it ideal for use in vaccines directed against intracellular pathogens, such as coccidial parasites (48). In fact, Quil A has been administered as a mucosal adjuvant against toxoplasmosis in subunit vaccines including as antigens crude rhoptry proteins of *T. gondii* (58, 59, 64) or recombinant ROP2 (54, 60). These formulations were evaluated in different host species, including cats (54, 58, 64), pigs (59) and mice (60), resulting in enhanced humoral (58–60) and cellular immune responses (59) but, at best, in partial protection against infection (59, 60, 64). In addition, i.p. immunization against murine toxoplasmosis using another recombinant antigen, *T. gondii* SAG3, resulted in increased survival rate and decreased cysts formation, through induction of a Th1-type immune response (63). Quil A was also used in an experimental vaccine against *N. caninum*, partially protecting mice against cerebral infection when s.c. co-administered with a *N. caninum* lysate (65). In a more recent research, Pastor-Fernández et al. (55) showed that i.p. immunization with different recombinant antigens from *N. caninum* (rNcROP4, rNcROP2, rNcGRA7, and rNcNTPasa) as monovalent or pair-wise combinations (rNcROP40 + rNcROP2 and rNcGRA7 + rNcNTPase) + Quil A, increased specific immune responses, decreased parasite burden in brain and partially protected against vertical transmission in a pregnant mouse model of congenital neosporosis.

**Table 1:** Plant compounds used as adjuvants in vaccine formulations against coccidial parasites.

**Type of compound**	**Adjuvant**	**Antigen/s**	**Pathogen**	**Animal model**	**Route of adm**.	**Results**	**References**
Saponins	Quil A	rRop2	*T. gondii*	DomesticCats	I.N.	Did not protect against oocyst shedding	(54)
Saponins	Quil A	rNcRop2,rNcRop4,rNcGra7,rNcNTPasa	*N. caninum*	PregnantBALB/c mice	I.P.	Increased pup survival and specific immune response	(55)
Saponins	QCDCQCDC RT	rProfilin	*E. acervulina*	BroilerChickens	S.C.	Increased body weight gain, CD4+/CD8+ and TCR1+/TCR2+ ratios and specific antibodies production. Decreased intestinal lesions	(56)
Saponins	QCDCQCDC R	rProfilin	*E. acervulina*	Chickens	S.C.	Increased body weight gain and mitogen-induced lymphocyte production. Decreased intestinal lesions. No effect on oocyst shedding	(57)
Saponins	Quil A	Crude rhoptry proteins	*T. gondii*	Cats	I.N.R.	Increased IgGs, no correlation with oocyst shedding	(58)
Saponins	Quil A	Crude rhoptry proteins	*T. gondii*	Pigs	I.N.	Increased local and systemic immune response (IgG, IgA, IgM). Conferred partial protection against brain cyst formation	(59)
Saponins	Quil A	rTgRop2	*T. gondii*	BALB/c mice	I.N.	Increased IgG, IgM, IgA and lymphoproliferation	(60)
Saponins	QCDC	rProfilin	*E. maxima*	ChickenEmbryos	(n.e.)	Increased body weight gain. Decreased oocyst shedding	(61)
Saponins	QCDC	rProfilin	*E. acervulina*	Broiler chickens	S.C.	Increased IgGs. No effect on oocyst shedding	(62)
Saponins	Quil A	rGST-SAG3	*T. gondii*	BALB/cmice	I.P.	Increased survival rate, IgG2a, number of TCD8+ cells, IFN-γ Mrna and nitric oxide release. Decreased number of cysts in brain tissue	(63)
Saponins	Quil A	Crude rhoptry proteins	*T. gondii*	Cats	I.N.	Decreased oocyst shedding	(64)
Saponins	Quil A	*N. caninum* tachyzoites or parasite lysate	*N. caninum*	BALB/cmice	S.C.	Decreased symptoms of cerebral neosporosis	(65)
Saponins	ISCOMs	rNcSAG1rNcHsp20rNcGRA7	*N. caninum*	Pregnant cattle	S.C.	Increased IgGs.Failed to prevent fetal infection	(66)
Saponins	ISCOMs	Native antigen extract from Nc-6 strain	*N. caninum*	Pregnant heifers	S.C.	Highly immunogenic, failed to prevent fetal infection	(67)
Saponins	ISCOMs	*N. caninum* live tachyzoites or native antigen extract	*N. caninum*	Cattle	S.C.	Increased total IgGs and IgG1No effect on IFN-γ productionNo effect on parasitaemia	(68)
Saponins	ISCOMs from native plants	*E. tenella* total antigens	*E. tenella*	Broiler chickens	I.N.	Increased IgGs Conferred protection against infection	(69)
Saponins	ISCOMs	Sporozoite antigens	*E. tenella*	Broiler chickens	I.N.	Decreased lesion score and oocyst shedding	(70)
Saponins	ISCOMs	rNcSRS2	*N. caninum*	BALB/c mice	S.C.	Decreased parasitaemia, parasite load in brain and clinical symptoms of disease	(71)
Saponins	ISCOMs	Crude rhoptry proteins	*T. gondii*	Pigs	S.C.	Partial protection against chronic infection	(72)
Saponins	ISCOMs	*T. gondii* native antigens	*T. gondii*	Sheep	S.C.	Increased IgG, IgA and antigen-stimulated lymphoproliferation	(73)
Saponins	ISCOMs	Sporozoite surface antigen (AgP27)	*E. falciformis*	BALB/c mice	O	Partial protection against oocyst challenge	(74)
PS	AstE, LenE, TreE	Live vaccine	*E. tenella*	Broiler chickens	D.S.	Increased total body weight gain. Conferred partial protection against infection	(75)
PS	Water-soluble extract of *A. membranaceus*	UV-attenuated *T. gondii*	*T. gondii*	ICR mice	I.P.	Increased Th1immune response and survival time, decreased parasite burden and histopathological score	(76)
PS	Providean-AVEC®	Total soluble antigens (SNcAg)	*N. caninum*	BALB/c mice	S.C.	Limited multiplication of the parasite. Activated dendritic cells and enhanced immune response	(77)
PS	Providean-AVEC®	Total soluble antigens (SNcAg)	*N. caninum*	Cattle	S.C.	Increased humoral and cellular immune response	(78)
Lectin	ScLLArtinM	Used as Chemotherapeutics	*T. gondii*	C57BL/6 mice	I.P.	Decreased parasite burden in brain. Increased secretion of cytokines and survival rate	(79)
Lectin	ScLL	Neospora lysate antigen (NLA)	*N. caninum*	C57BL/6 mice	S.C.	Increased IgG1, total IgGs and increased survival rate. Decreased parasite burden. No effect in IgG2a production	(80)
Lectin	ArtinMJacalin	Neospora lysate antigen (NLA)	*N. caninum*	C57BL/6 mice	S.C.	ArtinM+NLA increased total IgG and IgG2a/IgG1 ratio. Conferred partial protection from *N. caninum* infectionJacalin+NLA only mild effects, failed to protect mice against infection	(81)

*I.N., intranasal; I.P., intraperitoneal; S.C., subcutaneous; R, rectal; O, oral; D.S., diet supplement; PS, polysaccharides; AstE, Astragalus membranaceous extract; LenE, Lentinus edodes extract; TreE, Tremella fuciformis extract*.

In order to obtain a highly effective adjuvant, Quil A has also been formulated as adjuvant complexes. Among them, QCDC is composed by Quil A, cholesterol, dimethyl dioctadecyl ammonium bromide (DDA), and Carbopol (84), whereas the further incorporation of Bay R1005 [R], a synthetic glycolipid analog, endows the complex with the ability to trigger both Th1-and Th2-type immunity, giving the QCDCR adjuvant a broad range of desirable immune enhancing characteristics (84). QCDC has been evaluated as adjuvant in subunit recombinant vaccines containing Profilin (rProfilin) against avian coccidiosis, both *in ovo* immunization (61) and through the s.c. route in broiler chickens (62), with contradictory outcomes, probably as a result of the species of *Eimeria* used in each experimental infection (11) (Table 1). The same antigen (rProfilin) was also formulated with QCDCR and used in a s.c. immunization protocol in chickens, that after experimental infection with *E. acervulina* showed decreased intestinal lesions, increased body weight gain, and *ex-vivo* mitogen-induced lymphocyte proliferation (57). In addition, the incorporation of CpG ODN[T] to QCDCR (QCDCR-T) was able to increase the ratios CD4^+^/CD8^+^, TCR1^+^/TCR2^+^ and the serum antibody titers against rProfilin in s.c. vaccinated and *E. acervulina* challenged chickens (56).

On the other hand, saponins could also be formulated as immune-stimulatory complexes (ISCOMs), which are particulate antigen delivery systems composed of antigen, cholesterol, phospholipids and Quil A (48) or ISCOMATRIX™ vaccines, a similar formulation than ISCOMs but with much broader application (85). Unlike most other adjuvants, ISCOMs are able to elicit both CD8^+^ and CD4^+^ T cell responses in mammals (36) and are approved for veterinary vaccines (36, 86). In fact, Pinitkiatisakul et al. (71) demonstrated that s.c. immunization with recombinant protein SRS2 from *N. caninum* (rNcSRS2) + ISCOMs diminished the clinical symptoms of the disease in a mouse model of cerebral neosporosis. However, s.c. immunizations with vaccine formulations containing ISCOMs as adjuvants and native antigen extracts (67, 68) or the combination of recombinant *N. caninum* proteins (66) in the target species, *Bos taurus*, induced high titers of IgGs (66–68) and similar levels of IFN-γ to those achieved after inoculation with live NC-1 (68), but failed to prevent vertical transmission (66, 67). Similarly, examples of ISCOMs vaccine formulations are found against *T. gondii* infection in s.c. immunization protocols in pigs using as antigens total native antigens from *T. gondii* (73) or crude ropthry proteins (72), also depicting high humoral (72, 73) and cellular immune responses (73) but only partial protection against infection (87). Finally, these immunostimulatory complexes were also included in immunization protocols against avian coccidiosis. Just to name a few, early studies from Kazanji et al. (74) demonstrated that a native surface sporozoite protein purified from *E. falciformis* (AgP27) incorporated in ISCOMs induced the secretion of high levels of serum IgG, local IgA, enhanced the cellular immune response, triggered antigen-stimulated *ex-vivo* proliferation of T-lymphocytes and conferred partial protection in an orally immunized mice model of coccidiosis. Later, García et al. (70) showed that a similar formulation (ISCOMs + native sporozoites from *E. tenella*) diminished the intestinal lesions score and the oocyst shedding in *E. tenella* infected broiler chicken. In a more recent work, Berezin et al. (69) showed that i.n. immunized broiler chickens with formulations of ISCOMs containing purified saponins derived from native plants from Kazakhstan and *E. tenella* antigens, achieved significant immunostimulation and protection against challenge.

Although saponins obtained from *Quillaja saponaria*, such as Quil A and QS-21 and their derivative compounds, have proven adjuvant potential, their high toxicity and undesirable hemolytic effects have restricted their use in human vaccination (48). Therefore, considerable efforts have been made to discover new plant saponins with high adjuvant activity and reduced toxicity (48, 82, 83, 88, 89). Among them, saponins present in the leaves of *Quillaja brasiliensis*, especially a saponin fraction named QB-90, with remarkable structural similarities to Quil A, showed lower toxicity when subcutaneously administered to mice (88). In addition, QB-90 strongly potentiated the immune response to a viral antigen (*bovine herpes virus* type 1, BoHV-1), indicating that QB-90 is a safe and strong vaccine adjuvant (88). Many other alternative sources of saponins with immune-stimulatory properties and low toxicities include: saponins from the roots from *Panax notoginseng* (90), saponin fractions Rg1 and Rb1 from the root of *Panax ginseng* (91, 92), total saponins from stems and leaves of *P. ginseng* (87, 93), saponins from *Platycodon grandiflorum* (PGS) (94) and saponins from the roots of *Pulsatilla chinensis* (PCS) (89).

Up to now, the adjuvant activity of saponins has been related to its structure, which is comprised of hydrophilic sugar side chains and a hydrophobic aglycone back-bone (48). Nakaya et al. (95) suggested that the activity of adjuvant saponins would be initiated when saponins bind and activate specific receptors on APCs. In particular, ginseng extract stimulates the production of proinflammatory cytokines in macrophages via TLR4 (95). In addition, Bangham et al. (96) showed that Quil A is able to intercalate into cell membranes forming pores. This mechanism allows the antigen access to the antigen endogenous presentation pathway (96). However, it is unknown if those molecular mechanisms are common to most saponins. In the case of ISCOMS and ISCOMATRIX™ vaccines, they act as delivery system for most antigens, and their mechanism of action is very complex and combine antigen presentation by both MHCI and MHCII pathways, interaction with APC, stimulation of T helper subset, activation of CTLs and a broad immune response that depends on the induction of multiple immune mediators, which were extensively reviewed by Sun et al. (48). In this context, further investigation should be carried out to determine the possible modes of action exerted by saponins and their derivative compounds.

### Polysaccharides

**Figure 3:**
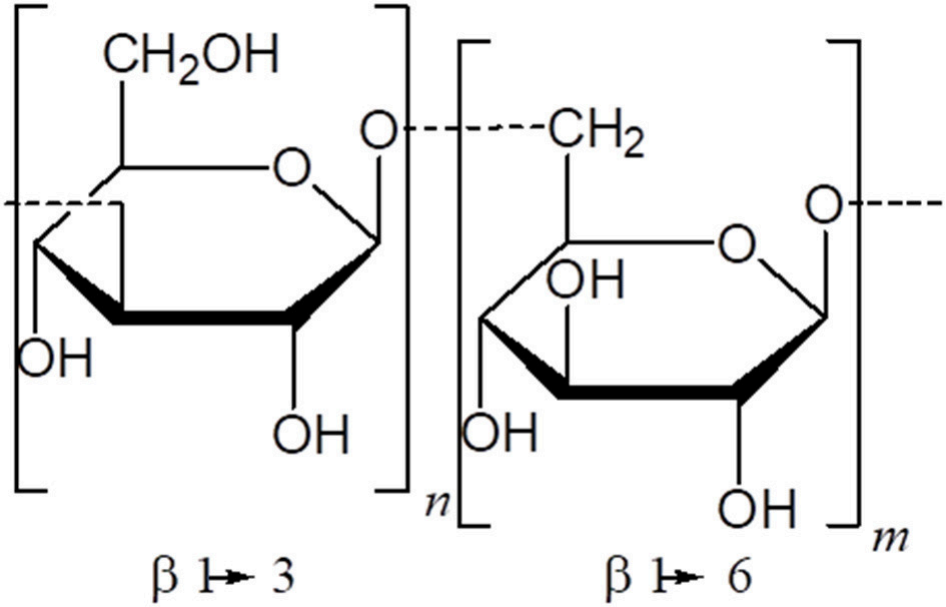
Common structural moiety of many bioactive polysaccharides.

Many polysaccharides from plants, and particularly, those derived from Chinese medicinal herbs, have emerged as excellent candidates to replace traditional adjuvants, since they can stimulate the immune system, are less toxic and have fewer collateral effects than bacterial polysaccharides and synthetic compounds (97) (Table 1). The common structural moiety of many bioactive polysaccharides from plants are basically, β-(1 → 6)-branched β-(1 → 3) gluco-oligosaccharides. Thus, the basic unit of β-glucan would have the immunostimulatory effects of the whole polysaccharide (98) (Figure 3).

In particular, polysaccharides from *Astragalus membranaceus* (AMPS) have shown important adjuvant capacity when added to vaccines against foot-and-mouth disease virus, infectious bursal virus, avian influenza virus and avian infectious bronchitis virus (IBV) [reviewed in (97)], demonstrating its potential for the development of vaccine formulations against intracellular pathogens such as Coccidia members. Indeed, a water-soluble polysaccharides extract from *A. membranaceus* have shown promising results against an experimental *E. tenella* infection in broiler chickens when fed with the extract as diet supplement and immunized with a live attenuated *E. tenella* vaccine (75). Moreover, Yang et al. (76) have shown that mice i.p. vaccinated with UV-attenuated *T. gondii* parasites co-administrated with water soluble extract of *A. membranaceus* exhibited longer survival rate, lower parasite burden, lower liver histopathological score, and higher Th1 response after challenge.

A new soy-lecithin adjuvant, Providean-AVEC®, was used by Mansilla et al. (77) in the development of a vaccine against neosporosis. This adjuvant, which contains β-glucans from barley and yeast and a soluble extract from *Chenopodium quinoa* whole seeds, has demonstrated to completely limit the multiplication of the parasite causing the pathogenesis, to activate DCs and to enhance cellular responses when formulated with soluble *N. caninum* antigens (SNcAg) and s.c. administered to mice (77), encouraging its use against this parasite. In a follow-up study, Mansilla et al. (78) demonstrated that the vaccine formulation containing Providean-AVEC® + SNcAg stimulated broad cellular and humoral immune responses against *N. caninum* in cattle. However, their effect on vertical transmission in heifers was not evaluated; thus, conclusions about vaccine efficacy were not conclusive.

To our knowledge, no other plant-derived polysaccharide has been used in vaccine formulations against coccidial parasites, despite their proven adjuvant potential (97). In fact, Advax™ is undoubtedly one of the most promising adjuvants obtained from plant sources, since it has been approved for human clinical trials (99, 100). Indeed, in a Phase 1/2 study in adult subjects, Advax™ adjuvant enhanced the immunogenicity of a recombinant hemagglutinin vaccine against pandemic influenza A/H1N1/2009 by increasing seroprotection rates with no adjuvant-associated adverse reactions observed (99). In addition, it also increased anti-Hepatitis B antigens (HBsAg) antibody titers and seroprotection rates when compared to administration of HBsAg alone in healthy human adults (100). Advax™ has also proven to successfully enhance vaccine immunogenicity across a broad range of antigen types and animal species tested so far, regardless of immunization routes, even when given during pregnancy or in early neonatal life [reviewed in (101)]. Hence, Advax™ arise as a candidate adjuvant for coccidial vaccines based upon its ability to stimulate both, CD8^+^ and CD4^+^ cell proliferation as well as Th1/Th2 cytokine response (102) and the characteristics mentioned above.

Among other plant polysaccharides with potential immunostimulatory properties is a water-soluble polysaccharide extracted from the roots of *Actinidia eriantha* (AEPS), a plant generally used in traditional Asian medicine (98). AEPS demonstrated to be a potent adjuvant for OVA-specific cellular and humoral immune responses, elicited a balanced Th1/Th2 immune profile in mice and caused neither mortality nor side effects when it was subcutaneously administered (98).

Finally, polysaccharides from the root of *Angelica sinensis* (ASPS), a well-known Chinese herbal medicine, have attracted much attention, since many studies have demonstrated that they have various bioactivities, such as hematopoiesis, immunomodulation, anti-oxidant and anti-tumoral effects [reviewed in (103)]. Besides, when incorporated as adjuvant in a Newcastle disease virus vaccine, it increased antibody titers, achieving better immune results than the vaccine alone (104).

All these examples show that plant polysaccharides are able to enhance specific responses against the antigen when administered parenterally. Also, plant polysaccharides have intrinsic muco-adhesive properties that may improve the interaction of the mucosa membrane with luminal antigen and facilitate its uptake, supporting the idea that plant polysaccharides can be used as oral adjuvants (105, 106). In this sense, Lemnan LM, apiogalacturonanic pectin of duckweed *Lemna minor*, was found to stimulate phagocytes and therefore tested, in the murine model, for adjuvant properties by oral administration with OVA protein antigen (105). Interestingly, the oral administration of the mixture of OVA and Lemnan achieved substantial systemic and local mucosal immune responses. Hence, Lemnan appears to elicit adjuvant activity via induction of both Th1 and Th2 responses (105). On the other hand, in mice, oral administration of poly phenylpropanoid-polysaccharide-rich extract of pine cones (PPC) suppresses the generation of IgE and enhances the generation of a Th1 cellular immune response (106), supporting the hypothesis that PPC could be used as oral adjuvant.

Although the mechanism of action of most plant polysaccharides remains elusive, it has been proposed that adjuvant activity starts with the binding to specific carbohydrate receptors expressed on APCs (107). In particular, macrophages might be activated by polysaccharides via TLR4, CD14, complement receptor 3 (CR3; also known as CD11b/CD18), scavenger receptors, dectin-1 and mannose receptor (108). The activation of these receptors leads to intracellular signaling cascades, resulting in transcriptional activation, monocyte maturation and the production of pro-inflammatory cytokines. In addition, activation of macrophages by polysaccharides can occur via an endocytosis-dependent pathway. Polysaccharides would become endocytosed after associating with macrophage receptors (101).

### Lectins

**Figure 4:**
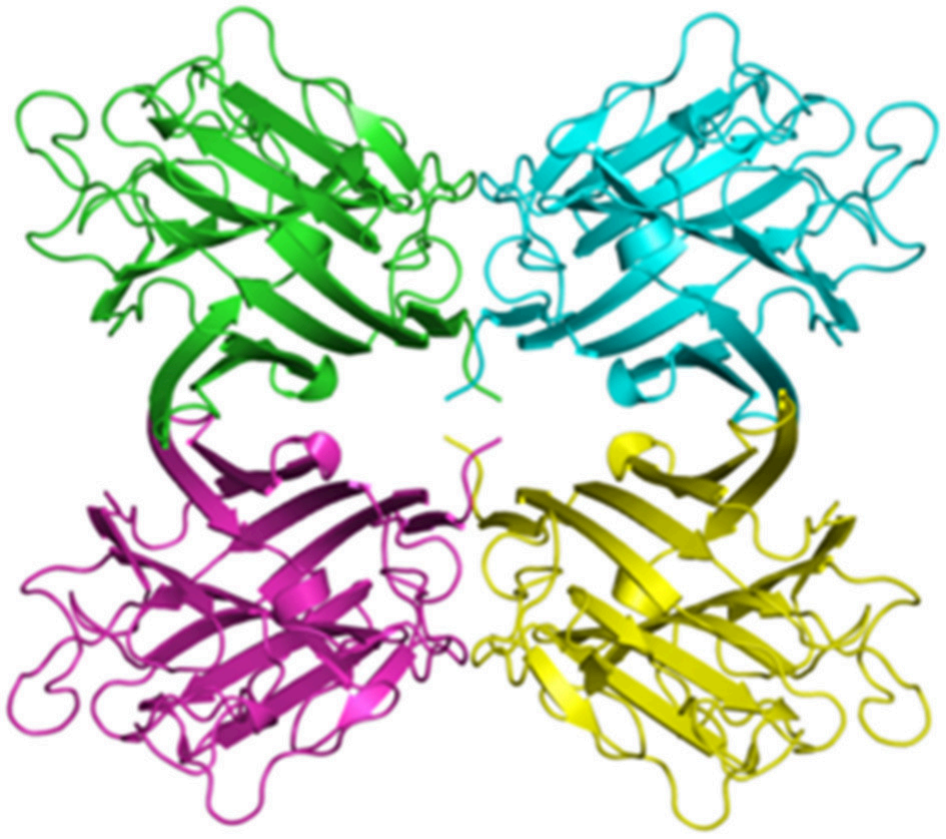
Homotetrameric assembly of Lectin UEA-II from *Ulex europaeus* (10.2210/pdb1dzq/pdb). This assembly was defined by Loris et al. (112). The crystal structure was determined using X-ray diffraction at a resolution of 2.85 Å and downloaded from the protein data bank: http://www.rcsb.org/.

Lectins exist in almost all living organisms and are carbohydrate-binding proteins which function as receptors to various cell surface glycoproteins, resulting in several important cell-mediated events, ranging from mitogenic processes to plant defense mechanisms (109, 110). In particular, plant lectins represent a biochemically and structurally varied group [for details see (111)], which probably reflects a certain degree of functional diversity (34). The main conformational component of most plant lectins is the β-sheet. Three main kinds of β-sheet architectures are found in plant lectins: the barrel, hevein domain and the jelly roll (or legume lectin fold) (110). The structure of a typical plant lectin is shown in Figure 4.

Plant lectins were first identified as proteins capable of agglutinating blood cells, and concanavalin A (ConA) from jack bean (*Canavalia ensiformis*) seeds was the first to be isolated almost a century ago (113). Several plant lectins showed immunomodulatory effects that are stimulated by their interaction with glycan's moieties present on the surface of immune cells (114). As a result of this interaction, signal transduction mechanisms are triggered to produce cytokines. Many plant lectins induce Th1 immunity [widely reviewed in (114)], manifested by high levels of IFN-γ production whereas a few stimulate Th2 immunity, as in the case of *Synadenium carinatum* latex lectin (ScLL) (115), leading to immune responses that could be beneficial against different pathogens and tumors.

The immunomodulatory properties of plant lectins have encouraged their screening for potential pharmaceutical applications, among them, the development of adjuvants. An important characteristic of certain plant lectins rely on their ability to interact with the mucosal epithelium and to be translocated across the gut, which may be exploited in vaccine formulations to induce mucosal and systemic immunity (47, 86) In the last few years, lectins from the jackfruit (*Artocarpus integrifolia*), ArtinM and Jacalin (JAC) have arisen as potential adjuvants in vaccines against protozoan parasites (80, 81, 116–119). In particular, ArtinM, stimulates macrophages and dendritic cells to produce IL-12 (120), through ArtinM interaction with the N-glycans of TLR2 (121), inducing a biased Th1-immune response. In fact, administration of ArtinM alone or in combination with soluble *Leishmania major* antigens (SLA) partially protects immunized mice against *L. major* (120) or *L. amazonensis* infection (116). The murine models of Leishmaniasis provide strong evidence for the immunomodulatory effect of ArtinM toward a Th1 profile through the modulation of IL-12 secretion. The beneficial effects of this lectin against *Leishmania* spp. have encouraged its evaluation as adjuvant/chemotherapeutic drug against coccidial parasites (Table 1). In fact, Cardoso et al. (81) have demonstrated that s.c. administration of ArtinM + Neospora lysate antigens (NLA) increased IgG and IgG2a/IgG1 ratio and partially protected mice from *N. caninum* infection. On the other hand, despite Jacalin, the major protein from *Artocarpus integrifolia* seeds, has demonstrated to act as a potent adjuvant when administered in combination with epimastigotes from *Trypanosoma cruzi* (117), only mild effects were obtained when s.c. administered to mice in combination with NLA against *N. caninum* (81).

Another plant lectin recently isolated and characterized by Souza et al. (122), a D-galactose-binding lectin named *Synadenium carinatum* latex lectin (ScLL), showed immunostimulatory, immunoprotective and adjuvant effects in a mouse model of cerebral neosporosis when administered with NLA, resulting in increased IgGs production, higher survival rate and decreased parasite burden (80) (Table 1). Peixoto Ferreira de Souza et al. (79) offered a different approach, and observed that i.p. treatment of mice with ScLL or ScLL + ArtinM previously infected with *T. gondii* significantly decreased parasite burden and increased survival rates, demonstrating the potential of ScLL and ArtinM lectins as immunotherapeutic agents against acute toxoplasmosis.

Although several plant lectins gathered the main characteristics of a potential adjuvant candidate to be used in vaccines against coccidial infections, most of them have not been evaluated in immunization protocols against these parasites yet. Among them, one of the best characterized is the B subunit of ricin toxin (RB) of the *Ricinus comunis*, which has been used as adjuvant/carrier protein fused to a reporter antigen (123), as well as to the simian rotavirus SA-11 non-structural protein NSP4 (124) in orally immunized mice, resulting in the secretion of IgG1 and IgG2 anti-GFP antibodies (123) or enhancement of Th1 immune response (124). Several other lectins, including lectins from *Viscum album* (mistletoe lectin 1; ML-1), *Lycopersicum esculentum* (tomato lectin; LEA), *Phaseolus vulgaris* (PHA), *Triticum vulgaris* (wheat germagglutinin (WGA), and *Ulex europaeus* (UEA-1) when evaluated as adjuvants in immunization protocols in mice, have demonstrated to stimulate the production of specific anti-bystander antigen (OVA) antibodies (serum IgG and IgA) (47). In particular, UEA-1 has proven to specifically bind to M cells from the Peyer's patches resulting in an excellent candidate for microencapsulation strategies (34). In fact, Manocha et al. (125) have demonstrated that i.n. immunization of mice with HIV peptides entrapped in polylactide-coglycolic acid microparticles in combination with UEA-I enhanced systemic and mucosal immune response. In addition, vaccine formulations containing killed whole *Helicobacter pylori* or *Campylobacter jejuni* conjugated to UEA-1 induced protective immune responses against live challenge in orally immunized mice (126).

Regarding the mechanism of actions of plant lectins, it has been shown that some of these lectins are able to interact with glycosylated TLR receptors on macrophages and/or DCs. In fact, several plant lectins might act as TLR agonists (118). The soybean (SBA), peanut agglutinin (PNA), ConA, and PHA lectins (PHA-L) are able to stimulate extracellular TLRs (118). In particular, Souza et al. (114), showed that ArtinM recognizes TLR2 N-glycans, but not TLR4 N-glycans. In addition, the production of IL-12 by macrophages under ArtinM stimulation requires the MyD88 adaptor molecule (114).

### Heat Shock Proteins

**Figure 5:**
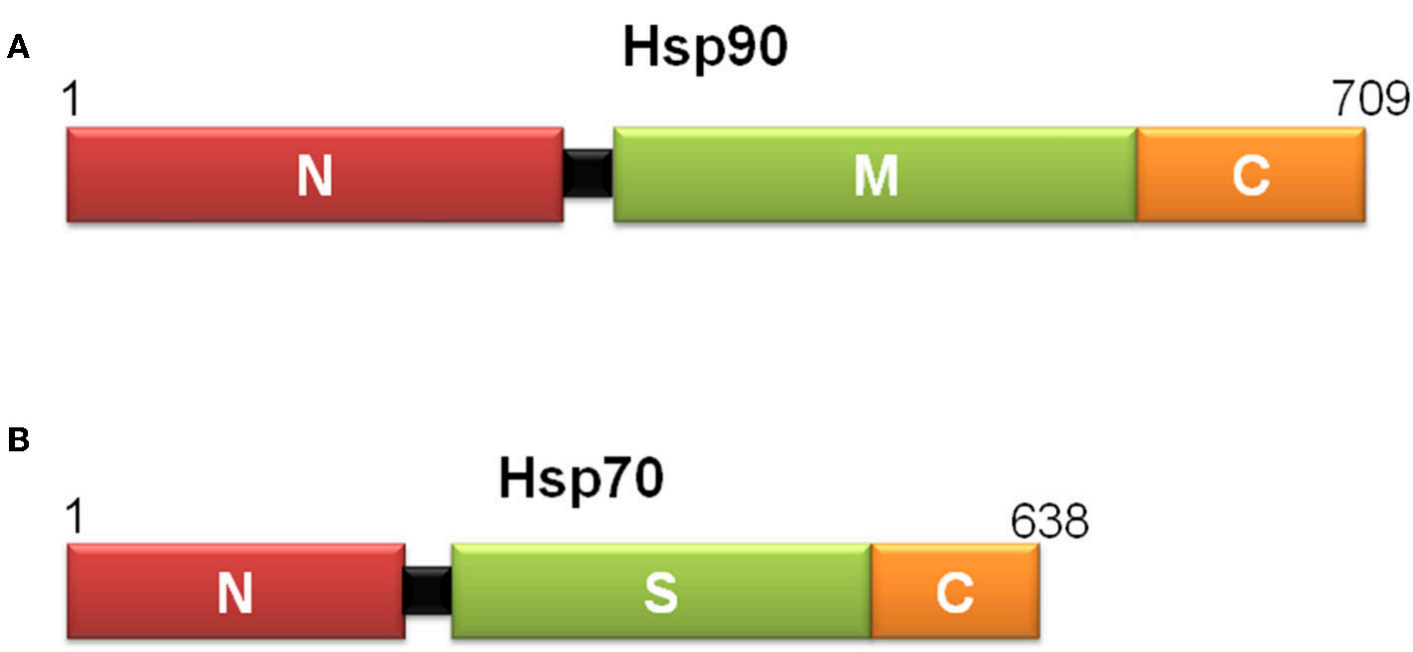
Scheme of the domain structure of yeast Hsp90 **(A)** and *E. coli* Hsp70 **(B)**, defined by limited proteolysis and structural studies. Hsp90s have an N-terminal peptide binding domain (N), a middle segment (M) that interacts with client proteins and contributes with ATP hydrolysis, and a C-terminal domain (C), involved in homodimerization. Hsp70s consist of an N-terminal ATPase domain (N) followed by a substrate binding domain (S) and a C-terminal domain (C), which forms a lid-like structure over the substrate-binding pocket that helps trap substrates in the substrate binding domain.

Heat shock proteins (Hsps) are molecular chaperones essential for preventing inappropriate associations or aggregations of partially folded proteins (127). They are highly conserved among eubacteria, yeasts, plants and animals (127). According to their molecular weights and degree of identity, Hsps are grouped in Hsp110, Hsp90, Hsp70, Hsp60, Hsp40, and small Hsps (128). Hsp90 has three structural domains: an N-terminal nucleotide binding domain that also binds Hsp90 inhibitors and may bind peptides; a middle segment that interacts with client proteins; and the C- terminus, which is involved in homodimerization (Figure 5A). In contrast, Hsp70 has two domains: an N-terminal nucleotide binding domain and a substrate binding domain (Figure 5B). For these two proteins, the structural basis of peptide binding and dynamic models of ligand interaction is notably understood, but how the basic biology of Hsps influences their immunological functions remains uncertain.

Early studies showed that Hsp70 or Hsp90 peptides complexes purified from different types of cancers were able to produce specific anti-tumor immunity (129, 130). Later, several researchers found that Hsps were able to bind antigenic peptides (131), facilitating antigen presentation by the Major histocompatibility complex class I (MHC I) (132), resulting in activation of CD8^+^ T cells (133, 134). The combined properties of Hsps to drive innate stimulation and deliver antigens to the APCs represent a link between innate and adaptative immune responses, thus, their natural adjuvant capacity is now being exploited in prophylactic vaccines against infectious diseases. In particular, Hsp complex (HSPC) vaccines are based on the enrichment of Hsps from bacteria along with its bound protein cargo and can potentially produce an effective vaccine without requiring the addition of an exogenous adjuvant, as it has been demonstrated for a tuberculosis vaccine based on HSPC from BCG (T-BioVax) in mice (135) and a vaccine against meningitis (MenBioVax) derived from heat-shocked protein-antigen complex against *Neisseria meningitidis* in humans (136). Other vaccine approaches include the administration of Hsps derived from several organisms mixed, complexed or fused to many different antigens and evaluated as adjuvants/carriers. In fact, several examples of recombinant Hsps from bacteria or human sources covalently linked to viral or protozoan antigens have demonstrated to enhance both humoral and cellular immune responses in murine models (137–139). Similar results were observed using *L. infantum* Hsp70 (LiHsp70) fused to maltose binding protein (MBP) as reporter antigen and evaluated in mice (140). Later, LiHsp83 was fused to *T. gondii* recombinant Rop2, and used as adjuvant-free vaccine formulation (foot-pad injection) in mice, causing predominance of specific IgG2a/IgG2c isotype and IFN-γ secretion, which in turn, conferred a remarkable resistance against toxoplasmosis (141). In addition, recent results from our laboratory demonstrated that LiHsp83 fused to *T. gondii* SAG1 and expressed in tobacco plants not only stimulated the production of specific anti-SAG1 IgGs and partially protected orally vaccinated mice from *T.gondii* infection, but also increased the level of *T. gondii* antigen accumulation in leaves (142). In fact, the ability of Hsps to chaperone peptides could provide stability to recombinant proteins, increasing the production yields and providing added value to plant based platforms, suggesting that Hsps could be used as novel carriers/adjuvants for vaccine antigen candidates to improve immunogenicity of recombinant antigens produced in plants.

These results suggest that several Hsps and not just those from the same species could be used as adjuvants/carriers in vaccine development against different pathogens that require a Th1 response to confer immunity. Although the immunological properties of Hsp70 and Hsp90 from humans and other organisms as bacteria and apicomplexan parasites are also present in their plant orthologs (35, 143, 144), the carrier/adjuvant properties of plant Hsps are less explored, even considering the advantage that plant Hsps are not derived from human pathogens. However, Buriani et al. (35) demonstrated that the structure of plant-derived Hsp70 (pHsp70) can be superimposed to the mammalian homolog and that, similarly to the mammalian counterpart, pHsp70–polypeptide complexes can activate the immune system. The same authors showed that pHsp70 purified from plant tissues transiently expressing the influenza A virus nucleoprotein is able to induce both the activation of MHC class I restricted polyclonal T-cell responses and antibody production in different mouse strains without the need of exogenous adjuvant (143). Similarly, in a recent report of our laboratory, Corigliano et al. (144) showed that *in vitro* incubation of splenocytes from naïve mice with recombinant plant Hsp90 (rpHsp90) elicited the expansion of CD19^+^ population. These results were supported by immunofluorescence analysis suggesting a direct effect of rpHsp90 on B cell proliferation. In the same study, it was demonstrated that stimulation of splenocytes with rpHsp90 was TLR-4 dependent since a low proliferation of spleen cells from C3H/HeJ mice, which have a point mutation in the cytoplasmic region of the TLR4 receptor, was detected. In a more recent study, Corigliano et al. (145) showed that i.p. immunization of mice with a fusion protein composed by pHsp90s and a reporter antigen induced a strong Th1 response along with a CD8^+^ cytotoxic cell response conferring immunity against the reporter antigen.

Taken together, these data imply that plant Hsps combine various advantageous properties, such as the capacity to bind antigenic peptides, deliver them to APC, exert immune-stimulatory effects, enhance strong Th1 response, and in the case of plant-based vaccines, results based on LiHsp83 suggest that pHsps probably should increase antigen accumulation in vegetal tissue, encouraging their use as carrier/adjuvants in vaccine formulations against coccidial infections, both in mucosal and parenteral immunizations.

## Concluding Remarks and Future Perspectives

Undoubtedly, coccidial infections are a major public health concern, also responsible for some of the most important veterinary diseases, leading to important economic losses in poultry and cattle industries. Although prophylactic vaccines emerge as the best approach to control coccidial parasites, the currently available vaccines are mostly based on live parasites, depicting serious issues related to vaccine safety. Despite subunit vaccines are a safer and more sustainable option, there is a reason why veterinary (or medical) medicine has not developed recombinant antigen vaccines for the prevention of every coccidial infections: they have not been effective enough to protect immunized individuals. This statement does not imply that such vaccines will never attain better results, since the application of omics -technologies during the last decades has improved our knowledge of effective immune responses in the hosts, as well as, the molecular basis in the host cell-parasite interaction, which provides important information that can be exploited for the rationale of vaccines design, including the selection of the most appropriate adjuvant. However, this is not an easy task. Although it is generally accepted that a proper adjuvant for vaccines against coccidial infections should allow the antigen processing and its presentation to the host immune system to enhance a strong cellular immune response, with IFN-γ secretion and reduced toxicity, each parasite has its own strategies to invade the host species, multiply, and escape the immune system, which represents singular challenges to overcome in the development of vaccine formulations.

Plant derived-adjuvants have, at least, two remarkable properties: first, most of them are relatively non-toxic and do not cause significant side effects, which are a major concern associated with synthetic compounds and second, they have proven to potentiate the immune response even when administered orally, making attractive the use of these compounds for the development of mucosal vaccines. In fact, some saponin-derived adjuvants, such as Quil A, are currently used in veterinary vaccines. Indeed, Quil A has demonstrated to improve immune protection when formulated in anti-coccidial vaccines through a Th1-biased immune response. In addition, ISCOMs have been widely used in experimental vaccines against *Eimeria* spp., *T. gondii*, and *N. caninum* with promising results, which turn them into clear candidates to be tested in vaccination trials in the next few years. As detailed above, certain plant polysaccharide extracts have been used in experimental live vaccines or in immunization protocols containing native antigens from coccidial parasites confirming their adjuvant properties. However, up to now, the most attractive adjuvant derived from plant polysaccharides is Advax™, used in human influenza pandemic vaccine, which has demonstrated to be highly immunogenic and safe which encourages its use in the development of vaccine formulations against coccidial diseases. In addition, lectins and their derivative biodegradable polymers show immunomodulatory activities and extensive animal studies demonstrate their safety. In particular, ArtinM and ScLL not only are potent adjuvants in immunization protocols, but also have proven to be potential chemotherapeutic drugs against *T. gondii*. More recently, plant heat shock proteins Hsp70 and Hsp90 demonstrated to enhance both humoral and cellular immune responses, with a clear Th1-biased profile and CD8^+^ T cells activation, arising as novel non-pathogenic adjuvant/carrier candidates.

Data here reviewed suggest that plant-derived adjuvants open the possibility to attain the main goal in adjuvant research: a safe and non-toxic adjuvant capable of strongly boosting and directing immune responses that could be incorporated into different vaccine formulations against coccidial diseases. However, it is difficult to decide which adjuvant represent the best option to be included in immunization protocols, since the lack of well-defined infection models for each coccidial parasite has rendered the comparison of results obtained in different reports a difficult undertaking. A challenge ahead is to establish harmonized animal models that mimic the infections of the target species to be used in immunization protocols, as well as, to define which parameters have to be evaluated in order to properly asses vaccine efficacy.

## Author Contributions

VS, MGC, and MC contributed conception and design of the review. VS wrote the first draft of the manuscript. MGC and MC wrote sections of the manuscript. All authors contributed to manuscript revision, read and approved the submitted version.

### Conflict of Interest Statement

The authors declare that the research was conducted in the absence of any commercial or financial relationships that could be construed as a potential conflict of interest.
